# Comparison of zwitterionic *N*-alkylaminomethanesulfonic acids to related compounds in the Good buffer series

**DOI:** 10.3762/bjoc.6.31

**Published:** 2010-04-01

**Authors:** Robert D Long, Newton P Hilliard, Suneel A Chhatre, Tatiana V Timofeeva, Andrey A Yakovenko, Daniel K Dei, Enoch A Mensah

**Affiliations:** 1 Eastern New Mexico University, Physical Sciences (Chemistry), Station 33, Portales, NM 88130; 2 New Mexico Highlands University, Department of Natural Sciences, Las Vegas, NM 87701

**Keywords:** aminomethanesulfonate buffers, Good buffer, morpholinomethanesulfonic acid, p*K*_a_ comparison, zwitterion

## Abstract

Several *N*-alkyl and *N*,*N*-dialkylaminomethanesulfonic acids were synthesized (as zwitterions and/or sodium salts) to be tested for utility as biological buffers at lower pH levels than existing Good buffer compounds (aminoalkanesulfonates with a minimum of two carbons between amine and sulfonic acid groups as originally described by Norman Good, and in common use as biological buffers). Our hypothesis was that a shorter carbon chain (one carbon) between the amino and sulfonic acid groups should lower the ammonium ion p*K*_a_ values. The alkylaminomethanesulfonate compounds were synthesized in aqueous solution by reaction of primary or secondary amines with formaldehyde/sodium hydrogensulfite addition compound. The p*K*_a_ values of the ammonium ions of this series of compounds (compared to existing Good buffers) was found to correlate well with the length of the carbon chain between the amino and sulfonate moeties, with a significant decrease in amine basicity in the aminomethanesulfonate compounds (p*K*_a_ decrease of 2 units or more compared to existing Good buffers). An exception was found for the 2-hydroxypiperazine series which shows only a small p*K*_a_ decrease, probably due to the site of protonation in this compound (as confirmed by X-ray crystal structure). X-ray crystallographic structures of two members of the series are reported. Several of these compounds have p*K*_a_ values that would indicate potential utility for buffering at pH levels below the normal physiological range (p*K*_a_ values in the range of 3 to 6 without aqueous solubility problems) – a range that is problematic for currently available Good buffers. Unfortunately, the alkylaminomethanesulfonates were found to degrade (with loss of their buffering ability) at pH levels below the p*K*_a_ value and were unstable at elevated temperature (as when autoclaving) – thus limiting their utility.

## Introduction

**Figure 1:**
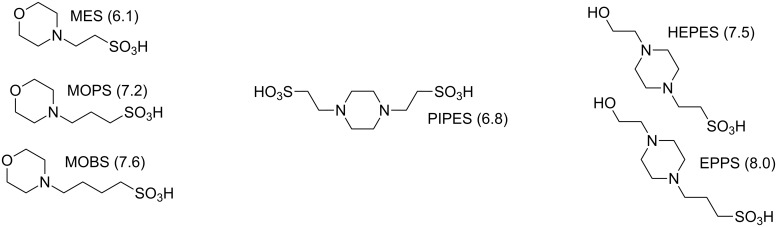
Examples of some currently available Good buffers (and their reported p*K*_a_ values) for the low end of the physiological pH range.

Some of the most widely used biological buffers are the compounds based on tertiary and secondary aminoalkanesulfonic acids (and their salts) initially reported by Norman Good, and which are now available from commercial sources (Figure 1) [1–2]. While these compounds provide good coverage of the physiological pH range and above, they have not been as widely useful in biochemical studies at acidic pH levels. Other classes of buffers that are usable in acidic pH ranges can be problematic in biochemical studies because of toxicity or metabolic interferences (i.e. citrate, borate, phosphate). This is particularly the case in studies of acidiophilic bacteria or biochemical systems that operate most efficiently in an acidic pH range. The impetus for this study was to investigate compounds with the potential for use as biological buffers in acidic pH ranges.

**Figure 2:**
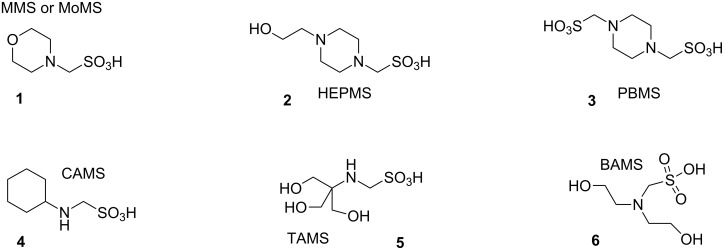
Aminomethanesulfonic acids in this study and their proposed acronyms.

Our focus was to synthesize a series of compounds that are analogous to currently available Good buffers – but with a single carbon atom between the amino and sulfonate functional groups (Figure 2). Our approach to the problem was based on noting that the p*K*_a_ values of currently available Good buffers decrease systematically as the number of carbon atoms decrease between the amine and sulfonic acid moieties [2–3]. We reasoned that by synthesizing compounds with a single carbon linkage (α-aminosulfonic acids) – we should be able to decrease the p*K*_a_ of the resulting compounds.

**Figure 3:**
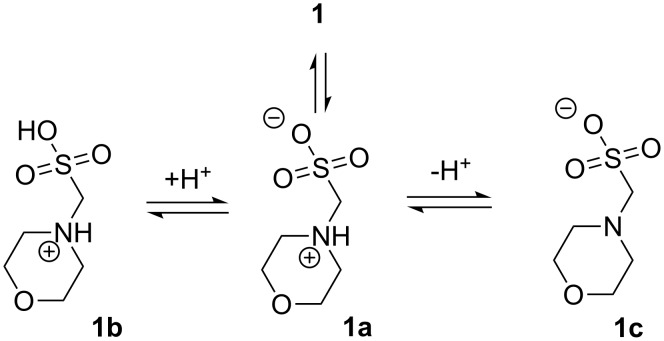
Possible ionization states of **1**.

The aminoalkylsulfonic acids are similar to amino acids, in that they can exist in multiple states of ionization, including a zwitterionic form (**1a**) at the isoelectric point (Figure 3). Since the ionization of the sulfonic acid functional group is essentially complete in aqueous solution, for purposes of creating buffers we are primarily interested in the p*K*_a_ of the ammonium ion proton dissociation (**1a** to **1c**). The p*K*_a_ of this dissociation is observed to be influenced by the proximity of the sulfonic acid group in the Good’s buffer class of compounds. In previous work on the series of unsubstituted aminosulfonates with 0–3 carbons between the amino and sulfonate groups, the p*K*_a_ for sulfamic acid, aminomethanesulfonic acid, and taurine (2-aminoethanesulfonic acid) in water are reported to be 1.01, 5.75, and 9.06, respectively [4]. The rapid decrease in p*K*_a_ in the series is likely due to inductive electron-withdrawing effect of the sulfonic acid group acting through fewer bonds as the carbon chain length is decreased. While an inductive effect is the most likely explanation for the observed ammonium ion acidity, the possibility of a through space intramolecular interaction (i.e. hydrogen-bonding or similar stabilization) of the proton with the sulfonate moiety cannot be discounted as a contributing factor.

## Results and Discussion

### Synthetic methodology

**Scheme 1:**
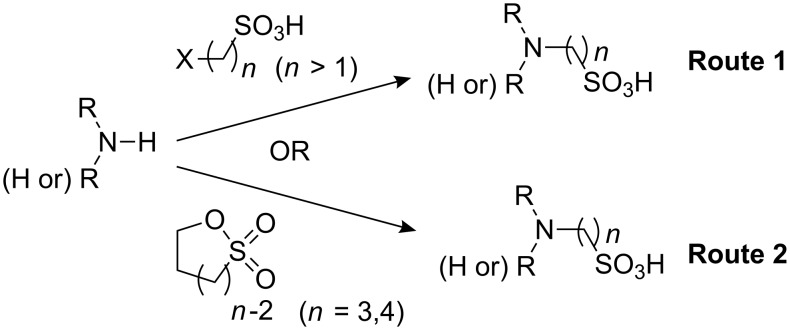
Routes reported previously for the synthesis of Good buffers.

Currently available Good buffers have been reportedly synthesized by one of two routes (Scheme 1). The first route is by nucleophilic substitution of haloalkylsulfonic acids (or their derivatives) with the selected primary or secondary amine, as described by Good [1–2]. The second route is by ring-opening of cyclic sulfonate esters (sultones) with an appropriate primary or secondary amine. However, this method is limited by the available sultone ring size to the production of 3 carbon aminoalkylsulfonates and larger [5].

**Scheme 2:**
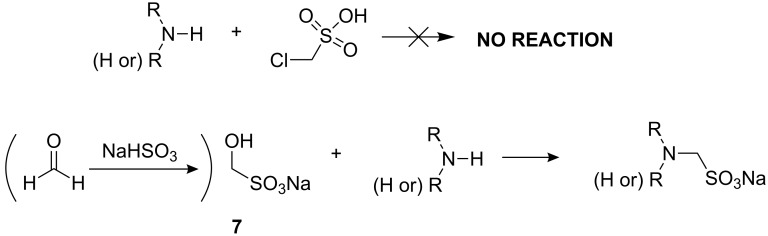
Synthetic routes investigated.

The second approach above is not amenable to the synthesis of aminomethanesulfonates, so our initial efforts were to synthesize the initial target compound *N*-morpholinomethanesulfonic acid (MMS) (**1**) by the reaction of morpholine with chloromethanesulfonic acid (Scheme 2). In spite of numerous attempts, this route produced no product, however. A subsequent literature search indicated that there were some prior reports of the lack of reactivity of chloro- and bromomethanesulfonic acids (and related derivatives) in nucleophilic substitution reactions, especially those with weaker nucleophiles such as amines [6–8]. We therefore abandoned this route in favor of a different approach previously reported in the literature for the specific synthesis of aminomethanesulfonates.

In this approach amines are reacted with the product of addition of sodium hydrogensulfite to formaldehyde [9–10]. The sodium hydrogensulfite/formaldehyde addition product **7** (sodium hydroxymethanesulfonate) is readily available from commercial sources and reacts in good yield with morpholine in aqueous solution over several days at room temperature, or in hours with moderate heating, to form the desired product **1** in a zwitterionic or salt form (depending on the workup conditions).

We extended this methodology to produce other compounds that are the α-aminosulfonic acid analogs of other commonly used Good buffers including *N*-(2-hydroxyethyl)piperazine-*N*′-methanesulfonic acid [HEPMS] (**2**), piperazine-*N*,*N*′-bis(methanesulfonic acid) [PBMS] (**3**), and cyclohexylaminomethanesulfonic acid [CAMS] (**4**). Our initial attempts to synthesize *N*-[tris(hydroxymethyl)methyl]-aminomethanesulfonic acid [TAMS] (**5**) have resulted in a product that is a mixture of the desired compound with what appears to be the O-alkylated derivative. We have also attempted to synthesize *N*,*N*-bis(2-hydroxyethyl)aminomethanesulfonic acid [BAMS] (**6**), but have been unable to characterize the product as it forms an intractable aqueous gel (hydrogel) that was completely resistant to all attempts to dehydrate it.

### Characterization as buffers

We then performed titrations to determine the p*K*_a_ values of proton dissociation from the ammonium ion (**1a** and equivalent). The p*K*_a_ values determined for these compounds, along with those of the analogous aminoethanesulfonate and aminopropanesulfonate buffer p*K*_a_ values for comparison is presented in Table 1.

**Table 1:** p*K*_a_ comparison of aminoalkylsulfonates. Acronyms refer to compounds in the Good’s buffer series that are commercially available. Some of the less common ones are CHES [2-(cyclohexylamino)ethanesulfonic acid], EPPS [4-(2-hydroxyethyl)-1-piperazinepropanesulfonic acid], PIPPS [piperazine-*N*,*N*′-bis(3-propanesulfonic acid], and CAPS [3-(cyclohexylamino)-1-propanesulfonic acid]. Experimental values reported at or above precision of measurement (see Supporting Information for statistics).

	p*K*_a_	Ethyl analog	p*K*_a_^a^	Propyl analog	p*K*_a_^a^

**1**	3.9	MES	6.2	MOPS	7.2
**2**	7.9	HEPES	7.5	EPPS	8.0
**3**	4.9^b^	PIPES	6.8^b^	PIPPS	8.0^b^
**4**	7^c^	CHES	9.3	CAPS	10.4

^a^Values from references [1,5,7]. ^b^2^nd^ (ammonium) dissociation constant value. ^c^Estimate only (precipitates at pH ≈ p*K*_a_).

As can be seen in Table 1, two of the new compounds (MMS **1** and PBMS **3**) have p*K*_a_ values that are significantly lower than their two and three carbon Good buffer counterparts (counting number of carbons between amine and sulfonic acid) and are well into the acidic pH range. All of the compounds except **2** show evidence of a systematic decrease in p*K*_a_ as the number of carbons between the amine and sulfonic acid functional groups decreases. In addition, PBMS **3** does not demonstrate the limited aqueous solubility that its two carbon analog PIPES does at low pH. CAMS **4** is the only one of the new compounds that shows limited solubility in aqueous solution (when pH ≤ p*K*_a_).

In order to assess the potential utility of these compounds as biological buffers, we conducted an initial experiment with different concentrations of the new compounds with *E. coli* strain HB101 at pH 7.2 and 37 °C for 18 h. Culture growth was estimated by optical density measurement at 600 nm, and the results are summarized in Table 2 (in which each value is the result of 3 separate trials, with an overall standard error for all final measurements of 0.025).

**Table 2:** OD_600_ of cultures with varying concentrations of aminomethanesulfonate.

Buffer	Initial OD_600_	Final OD_600_		

Conc. used		10 mM	20 mM	50 mM
**1**	0.015	1.16	1.22	1.18
**2**	0.015	1.16	1.08	0.66
**3**	0.015	1.23	1.21	1.08
Control	0.015	1.24	1.28	1.33

The results of this testing indicate that the three new buffer compounds (**1**, **2** and **3**) supported culture growth at all concentrations tested. However, there is some indication of possible toxicity or other culture growth inhibition occurring at the higher concentrations of HEPMS **2** and PBMS **3** due to the significantly lower optical densities resulting when the concentrations of these buffers were increased.

During testing it became apparent that these compounds are unstable at pH values that are below the p*K*_a_ value. They can stably maintain buffered pH at room temperatures for days when above their p*K*_a_ values, but at pH levels below the p*K*_a_ they rapidly lose the ability to maintain a buffering effect (pH drifts to higher values). The compounds also become unstable at elevated temperatures. When autoclaved, buffered solutions change pH and lose buffering capability. Therefore, while they may be suitable for niche use in some applications as lower pH biological buffers at room temperature, they are severely limited by the stability of the aminomethanesulfonate structure at low pH or elevated temperature.

### Crystallographic structures

**Figure 4:**
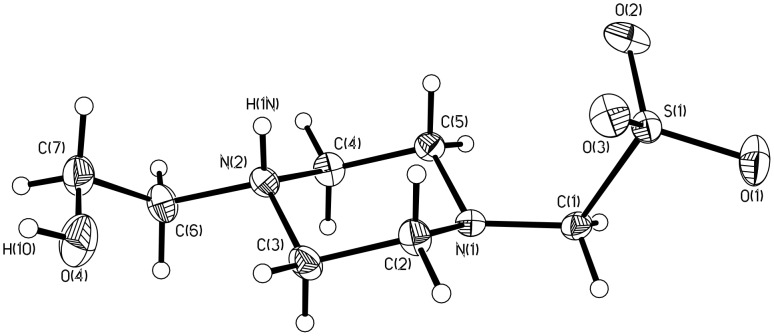
Crystal structure of HEPMS **2** with displacement ellipsoids at the 30% probability level.

**Figure 5:**
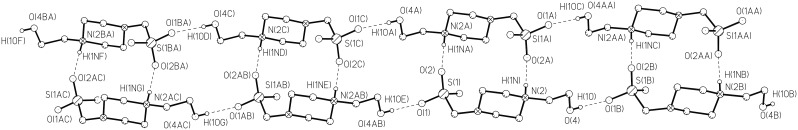
Hydrogen bonded molecular ribbon in solid phase **2** along [101].

All of the buffers exhibit a marked trend of p*K*_a_ decrease in Table 1, with the exception of the 2-hydroxypiperazine series (**2**, HEPES, and EPPS). A possible explanation of why this series is not as impacted by the shortening of the carbon chain between the sulfonic acid and amino group is if the zwitterion is formed by protonation at the piperazine nitrogen that is bonded to the hydroxyethyl functional group. This nitrogen’s basicity would be expected to be only slightly impacted by structural modifications of the alkylsulfonic acid group at the other nitrogen. This hypothesis is supported by the crystal structure of HEPMS **2** as a zwitterion shown in Figure 4. The crystal structure of **2** is consistent with the one previously reported for HEPES [11]. Both have piperazine rings in the chair conformation, with extended conformation for the side chains. Both have extensive intermolecular hydrogen bonding forming centrosymmetric dimers, although HEPMS **2** forms ribbons (Figure 5), while HEPES forms corrugated sheets.

The crystal structure of **2** shows protonation occurring at the hydroxyethylamino nitrogen (N2), which is consistent with the protonation found in the crystal structure of zwitterionic HEPES. Protonation at this site is likely favorable due to the regular hydrogen bonding formed in the solid phase (Figure 5). However, it is also likely that this nitrogen is protonated first in aqueous solution phase, as this nitrogen would be expected to be more basic than the aminomethanesulfonate nitrogen (N1).

In the other compounds, the only amine available for protonation is the amine attached to an alkylsulfonic acid group. Therefore, in these compounds (**1**, **3**, **4**) we see a pronounced reduction in the basicity of the amine as the carbon chain between the amine and sulfonic acid group has been reduced. As the basicity of the amine is reduced, the p*K*_a_ of its ammonium form is reduced – making it available for buffering in a lower pH range.

## Experimental

### Acid dissociation constants

Acid dissociation constants for the ammonium ions were determined by aqueous solution titrimetry (min. 5 repetitions from low to high, then high to low pH) using standardized NaOH and HCl solutions.

### General synthetic procedure

The compounds were prepared by dissolving 0.200 mol (26.8 g) of sodium hydrogensulfite-formaldehyde addition product in 250 mL deionized water in a 500 mL RBF with stirring. One equivalent (one-half equivalent in the case of piperazine) of the appropriate amine (0.200 mol) was then dissolved separately in 100 mL of deionized water and added to the stirred solution drop-wise via an addition funnel. The mixture was then stirred at 60 °C for 48 h. The volume of the reaction solution was reduced in vacuo by one-third, and then stored at 4 °C overnight to crystallize/precipitate the product. The precipitate was then collected by vacuum filtration and washed once with cold ethanol. Recrystallization solvents were limited to water and/or hot low M.W. alcohols (i.e. methanol, ethanol), as the materials are insoluble in other solvents.

***N*-Morpholinomethanesulfonic acid [MMS (or MoMS)] (1).** This was prepared as the half sodium salt by the general procedure from 0.200 mol of morpholine (17.4 mL), yielding 31.6 g (0.164 mol) of **1** (82% isolated yield) as a yellowish-white semi-crystalline material. The product was recrystallized from 50% ethanol-water solution. The p*K*_a_ of a 0.3 M solution at 20 °C was determined to be 3.8. mp 143–149 °C dec; IR (KBr) 3409(s), 3138(br), 1196, 1216(s), 1250, 1108, 1059, 1014 cm^−1^; ^1^H NMR (500 MHz, D_2_O/TSP): δ 2.92 (4H, t, *J* = 4.8), 3.76 (4H, t, *J* = 4.8), 3.8 (2H, s); ^13^C NMR (125 MHz, D_2_O/TSP): δ 54.7 (t), 69.2 (t), 75.8 (s). Anal. Calcd for C_5_H_11_NO_4_S·0.5Na: C, 31.24%; H, 5.51%; N, 7.29%; Na, 5.98%; O, 33.30%; S, 16.68%. Found: C, 31.05%; H, 6.13%; N, 7.63%; S, 16.66%. Structure assignment supported by single crystal X-ray structure of the sodium salt form (in Supporting Information File 1).

***N*-(2-hydroxyethyl)piperazine-*N*′-methanesulfonic acid [HEPMS] (2).** This was prepared as the sodium salt monohydrate by the general procedure from 0.200 mol of 1-(2-hydroxyethyl)piperazine (24.5 mL), yielding 49.2 g (0.186 mol) of **2** (93% isolated yield) as a white semi-crystalline solid. The product was recrystallized from ethanol. The p*K*_a_ of a 0.25M solution at 20 °C was determined to be 7.4. mp 178–185 °C dec; IR (KBr) 3408(s), 3240(br), 1223(s), 1163, 1057 cm^−1^; ^1^H NMR (250 MHz, D_2_O): δ 2.58–2.66 (6H, m), 2.96 (4H, t, *J* = 5.2), 3.76 (2H, t, *J* = 6.3), 3.81 (2H, s). ^13^C NMR (62.9 MHz, MeOD): δ 52.5, 54.0, 59.2, 60.7, 74.2. Anal. Calcd for C_7_H_17_N_2_NaO_5_S·H_2_O: C, 31.81%; H, 6.48%; N, 10.60%; Na, 8.70%; O, 30.27%; S, 12.13%. Found: C, 30.72%; H, 6.48%; N, 10.22%; S, 10.82%. Structure assignment supported by single crystal X-ray structure of the zwitterion form (in Supporting Information File 2).

**Piperazine-*N*,*N*′-bis(methanesulfonic acid) [PBMS] (3).** This was prepared as the disodium salt dihydrate by modification of the general procedure [using 0.400 mol (53.6 g) of formaldehyde-sodium hydrogensulfite addition product] from 0.200 mol of piperazine (17.2 g), yielding 46.5 g (0.131 mol) of **3** (66% isolated yield) as a white semi-crystalline solid. The product was recrystallized from 50% ethanol-water solution. The p*K*_a_ of a 0.3 M solution at 20 °C was determined to be 4.8. mp 226–245 °C dec; IR (KBr) 3400(s), 3144(br), 1244, 1223(s), 1190, 1059 cm^−1^; ^1^H NMR (500 MHz, D_2_O/TSP): δ 2.94 (8H, s), 3.80 (4H, s). ^13^C NMR (125 MHz, D_2_O/TSP): δ 54.1, 75.6. Anal. Calcd for C_6_H_16_N_2_Na_2_O_8_S_2_·2H_2_O: C, 20.34%; H, 4.55%; N, 7.91%; Na, 12.98%; O, 36.13%; S, 18.10%. Found: C, 20.28%; H, 4.32%; N, 8.33%; S, 16.59%.

**Cyclohexylaminomethanesulfonic acid [CAMS] (4).** This was prepared as the sodium salt by the general procedure from 0.200 mol of freshly distilled cyclohexylamine (22.9 mL), yielding 36.2 g (0.168 mol) of **4** (84% isolated yield) as white semi-crystalline powder. The product was recrystallized from 50% ethanol-water solution. The p*K*_a_ of 0.3 M solution at 20 °C was determined to be 7.8. mp 182–195 °C dec; IR (KBr) 3450(br), 3034(s), 1602, 1458, 1249, 1158, 1053 cm^−1^; ^1^H NMR (500 MHz, D_2_O/TSP): δ 1.14–1.22 (1H, m), 1.28–1.40 (4H, m), 1.63–1.69 (1H, m), 1.77–1.83 (2H, m), 1.97–2.02 (2H, m), 3.12–3.19 (1H, m), 4.40 (2H, s). ^13^C NMR (125 MHz, D_2_O/TSP): δ 26.7, 27.0, 33.2, 53.2, 77.0. Anal. Calcd for C_7_H_14_NNaO_3_S: C, 39.06%; H, 6.56%; N, 6.51%; Na, 10.68%; O, 22.30%; S, 14.90. Found: C, 39.56%; H, 7.05%; N, 7.29%; S, 15.95%.

## Supporting Information

^1^H and ^13^C NMR of compounds **1**–**5**, plus some 2D NMR spectra. Titration curves of compounds **1**–**3** and **5** and statistics from the titrations. Details of additional experimental protocols used. CIF files for MMS **1** and HEPMS **2**.

File 1 NMR spectra, titration curves, and additional experimental protocols

File 2 CIF file for MMS **1**

File 3 CIF file for HEPMS **2**
