# Supplementary material for: Comparison of zwitterionic N-alkylaminomethanesulfonic acids to related compounds in the Good buffer series
Source: Beilstein J Org Chem. 2010 Apr 1;6:31. doi: 10.3762/bjoc.6.31 (PMC2874393; doi:10.3762/bjoc.6.31)
Supplement: File 1 — NMR spectra, titration curves, and additional experimental protocols [file Beilstein_J_Org_Chem-06-31-s001.pdf]

Supporting Information for

Comparison of zwitterionic *N*-alkylaminomethanesulfonic acids to related compounds in the Good buffer series

Robert D. Long*[1], Newton P. Hilliard, Jr.[1], Suneel A. Chhatre[1], Tatiana V. Timofeeva[2], Andrey A. Yakovenko[2], Daniel K. Dei[1] and Enoch A. Mensah[1]

Address: [1]Eastern New Mexico University, Physical Sciences (Chemistry), Station 33, Portales, NM 88130 and [2]New Mexico Highlands University, Dept. of Natural Sciences, Las Vegas, NM 87701

Email: Robert D. Long - robert.long@enmu.edu

* Corresponding author

Table of Contents

Protocols and Methods	S3
500 MHz ^{1}H NMR of MMS **1** in D_2O	S4
125 MHz ^{13}C NMR of MMS **1** in D_2O	S5
gHMQC of MMS **1** on 500 MHz NMR	S6
250 MHz ^{1}H NMR of HEPMS **2** in D_2O	S7
63 MHz ^{13}C NMR of HEPMS **2** in MeOD	S8
500 MHz ^{1}H NMR of PBMS **3** in D_2O	S9
125 MHz ^{13}C NMR of PBMS **3** in D_2O	S10
500 MHz ^{1}H NMR of CAMS **4** in D_2O	S11
125 MHz ^{13}C NMR of CAMS **4** in D_2O	S12
COSY of CAMS **4** in D_2O on 500 MHz NMR	S13
gHMQC of CAMS **4** in D_2O on 500 MHz NMR	S14
500 MHz ^{1}H NMR of TAMS **5** in D_2O (with byproduct)	S15
125 MHz ^{13}C NMR of TAMS **5** in D_2O (with byproduct)	S16
gHMQC of TAMS **5** sample in D_2O (with byproduct)	S17
Titration curve of MMS **1**	S18
Titration curve of HEPMS **2**	S19
Titration curve of PBMS **3**	S20
Titration curve of (impure) TAMS **5**	S21
Statistics from Titrations data	S22
ORTEP figures of MMS **1** and HEPMS **2**	S23

Additional supporting information (in separate files): S2.CIF, S3.CIF

Protocols and methods

Optical absorbance spectra of 0.2 M solutions were recorded from 700 to 200 nm using a Perkin Elmer Lambda 35 dual beam spectrophotometer. Elemental composition was determined by combustion analysis at Texas Tech University, Lubbock, TX, USA. Melting points were determined using a Stanford Research Systems MPA-100 Optimelt melting point apparatus and are uncorrected. Infrared absorption spectra were recorded as KBr pellets using a Nicolet Magna 560 FT-IR. X-ray crystallographic analysis of MMS **1** was obtained by Robert Long during an ACS/PRF workshop entitled “X-ray Crystallography for Organic Chemists” at UCSD in Aug 2004. X-ray crystallographic analysis of HEPMS **2** was conducted by Tatiana Timofeeva and associates of New Mexico Highlands University. Crystals for X-ray analysis were obtained by recrystallization from saturated methanol solutions of the products. NMR analyses were conducted on 250 and 500 MHz instruments at Texas Tech University and University of New Mexico.

Growth Study protocol. *Escherichia coli* strain HB101 was cultured overnight in LB media at 37 °C with shaking. Filter sterilized stock solutions were made for each buffer. Each stock solution was adjusted to pH 7.2 (the pH of LB media) prior to sterilizing. Sterile buffer was added to culture tubes containing fresh LB media to final concentrations of 10, 20 and 50 mM. Each tube was inoculated from the overnight culture and shaken at 37 °C for ~18 h. Culture growth was estimated by measuring the optical density at 600 nm.

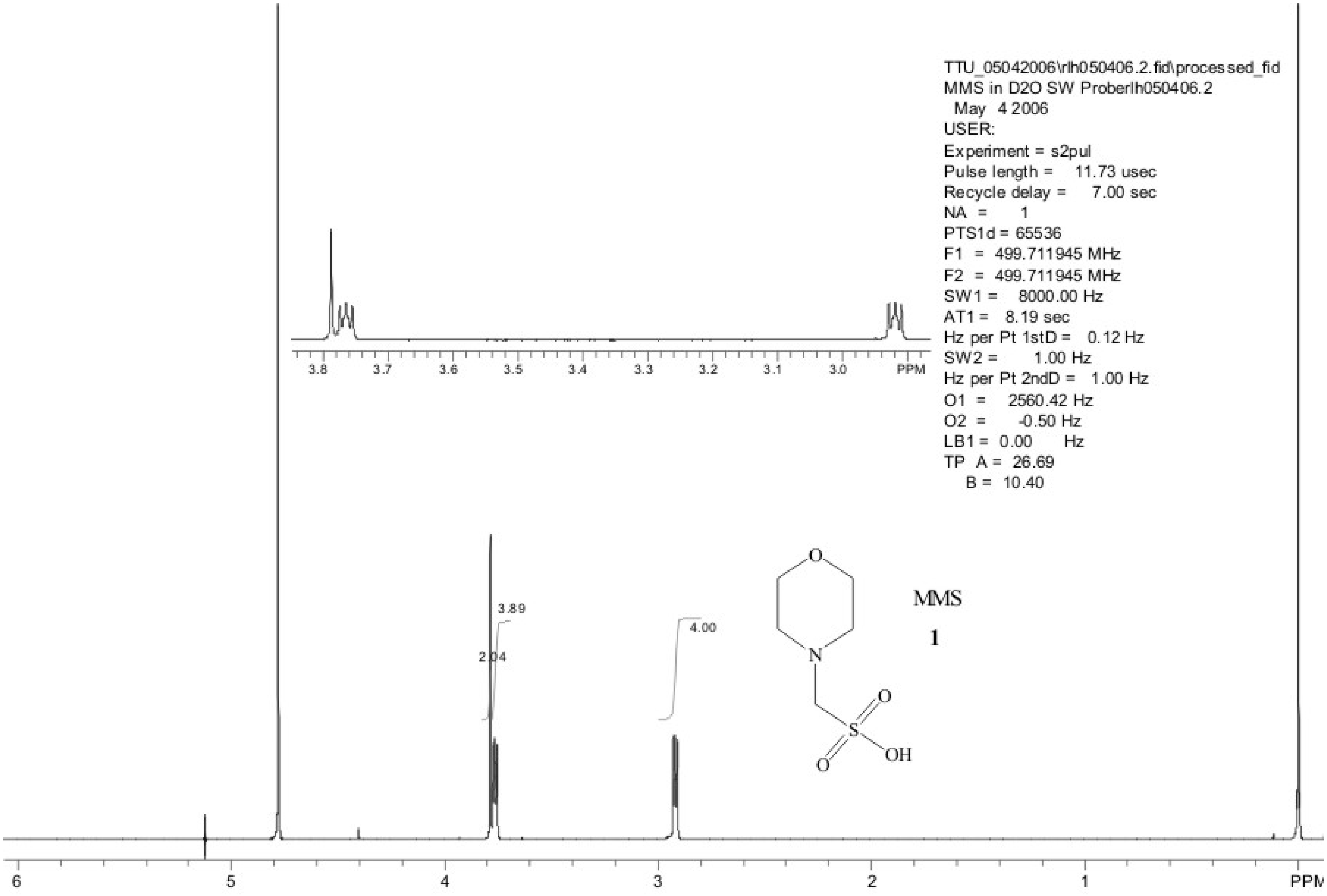
TTU_05042006\rlh050406.2.fid\processed_fid
MMS in D2O SW Proberlh050406.2
May 4 2006
USER:
Experiment = s2pul
Pulse length = 11.73 usec
Recycle delay = 7.00 sec
NA = 1
PTS1d = 65536
F1 = 499.711945 MHz
F2 = 499.711945 MHz
SW1 = 8000.00 Hz
AT1 = 8.19 sec
Hz per Pt 1stD = 0.12 Hz
SW2 = 1.00 Hz
Hz per Pt 2ndD = 1.00 Hz
O1 = 2560.42 Hz
O2 = -0.50 Hz
LB1 = 0.00 Hz
TP A = 26.69
B = 10.40
MMS
1
3.89
2.04
4.00
PPM
6
5
4
3
2
1
3.8
3.7
3.6
3.5
3.4
3.3
3.2
3.1
3.0

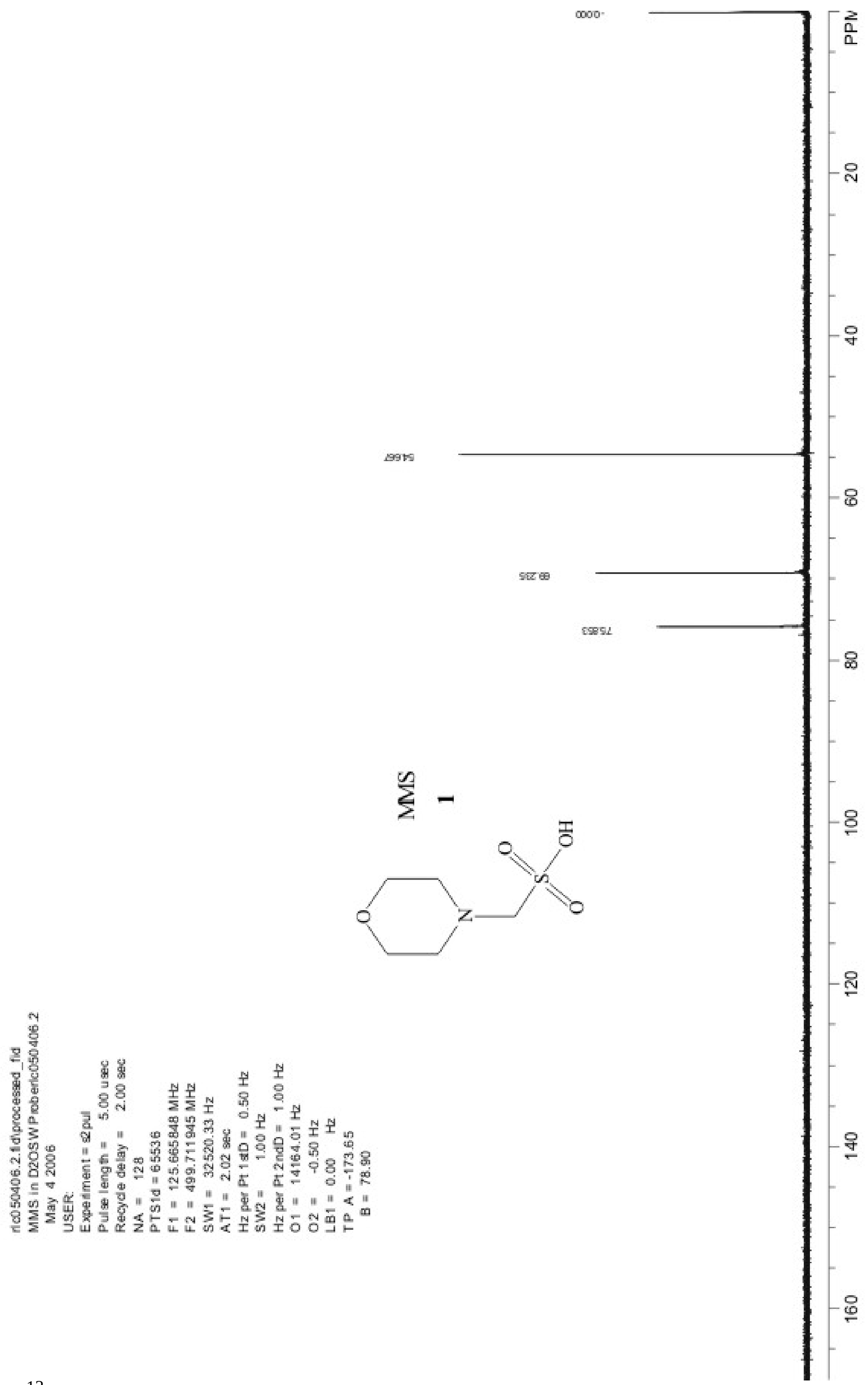

125 MHz ^{13}C NMR of MMS **1** in D_2O with TSP reference

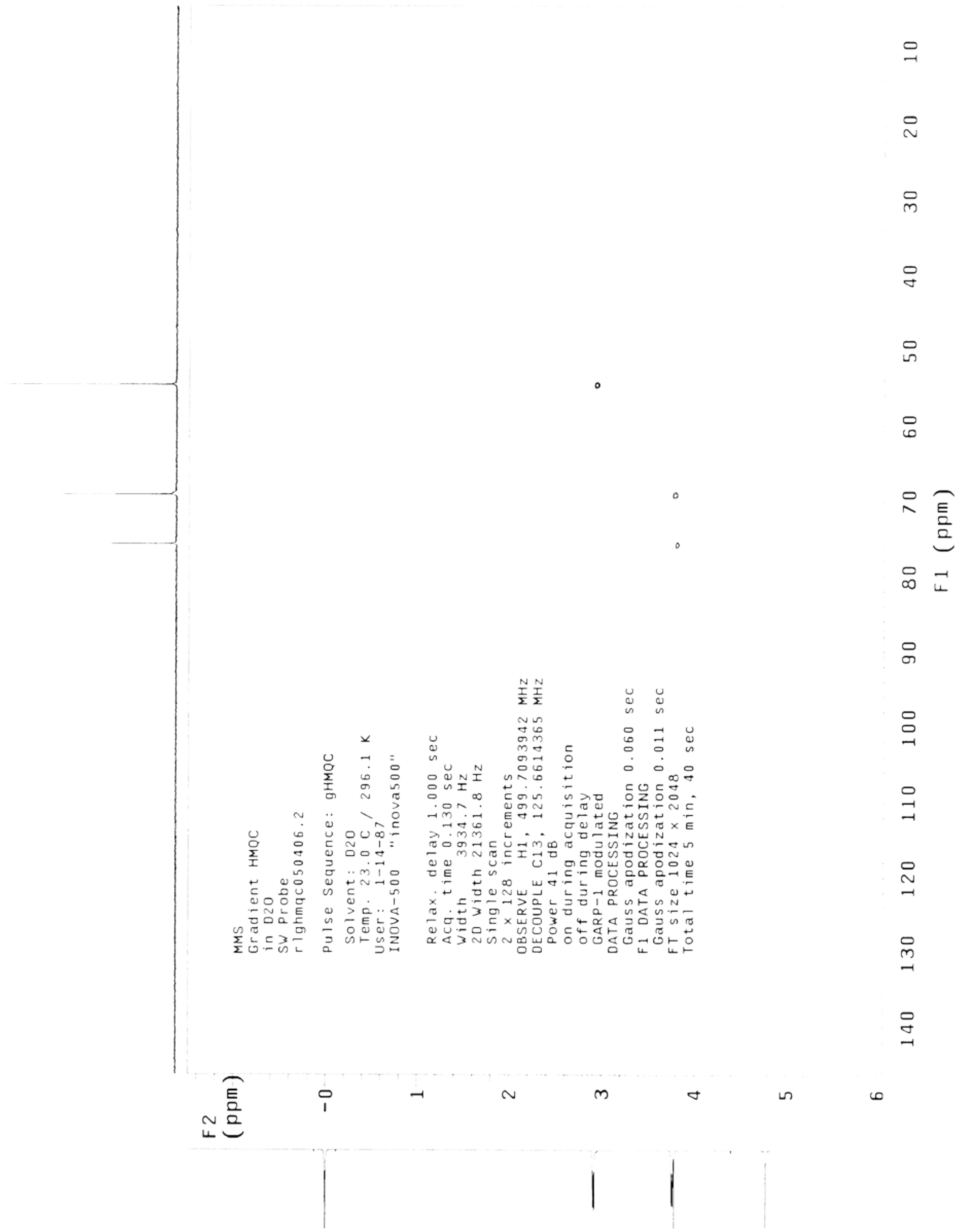

gHMQC of MMS **1** in D_2O on 500 MHz NMR

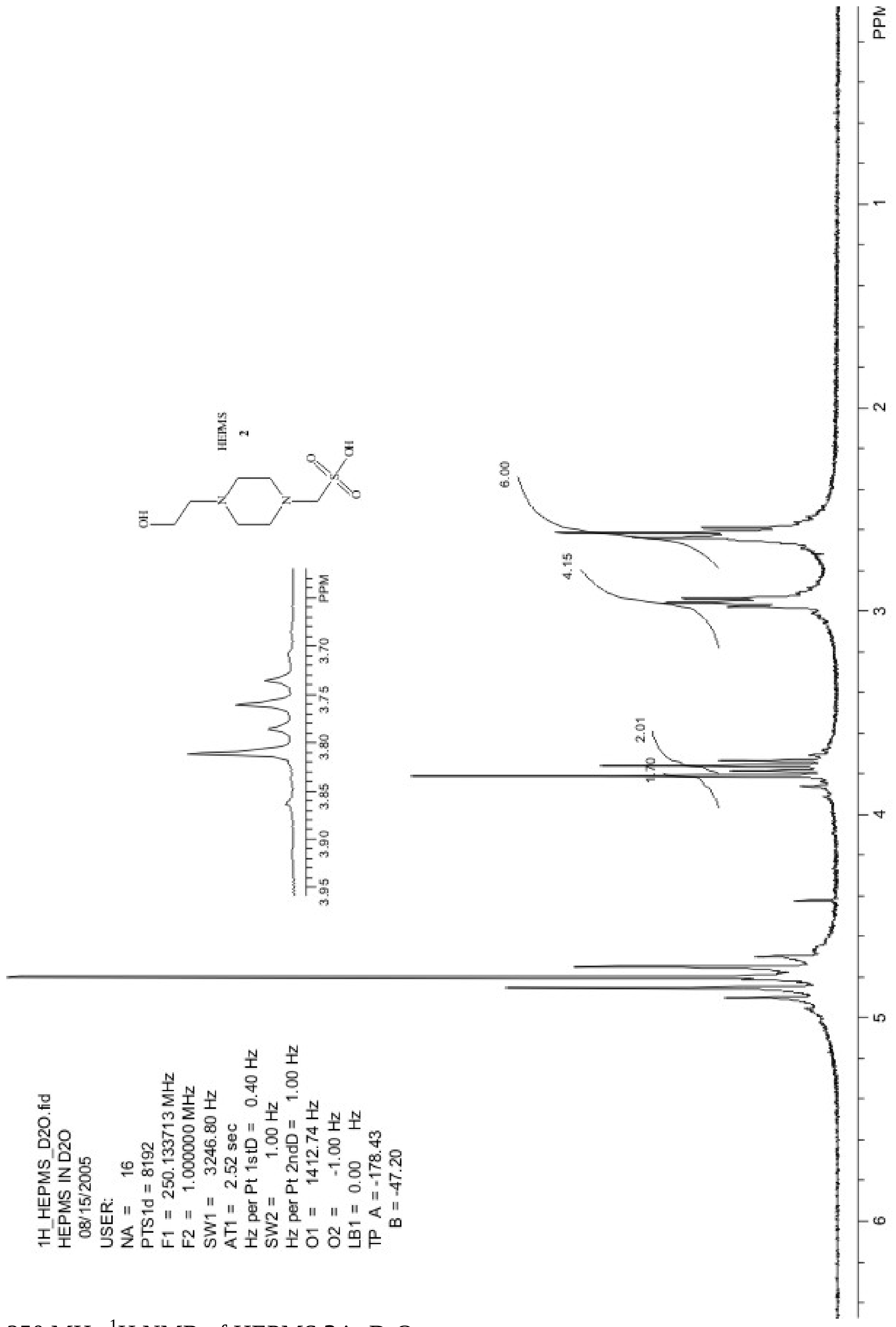

250 MHz ^{1}H NMR of HEPMS **2** in D_2O

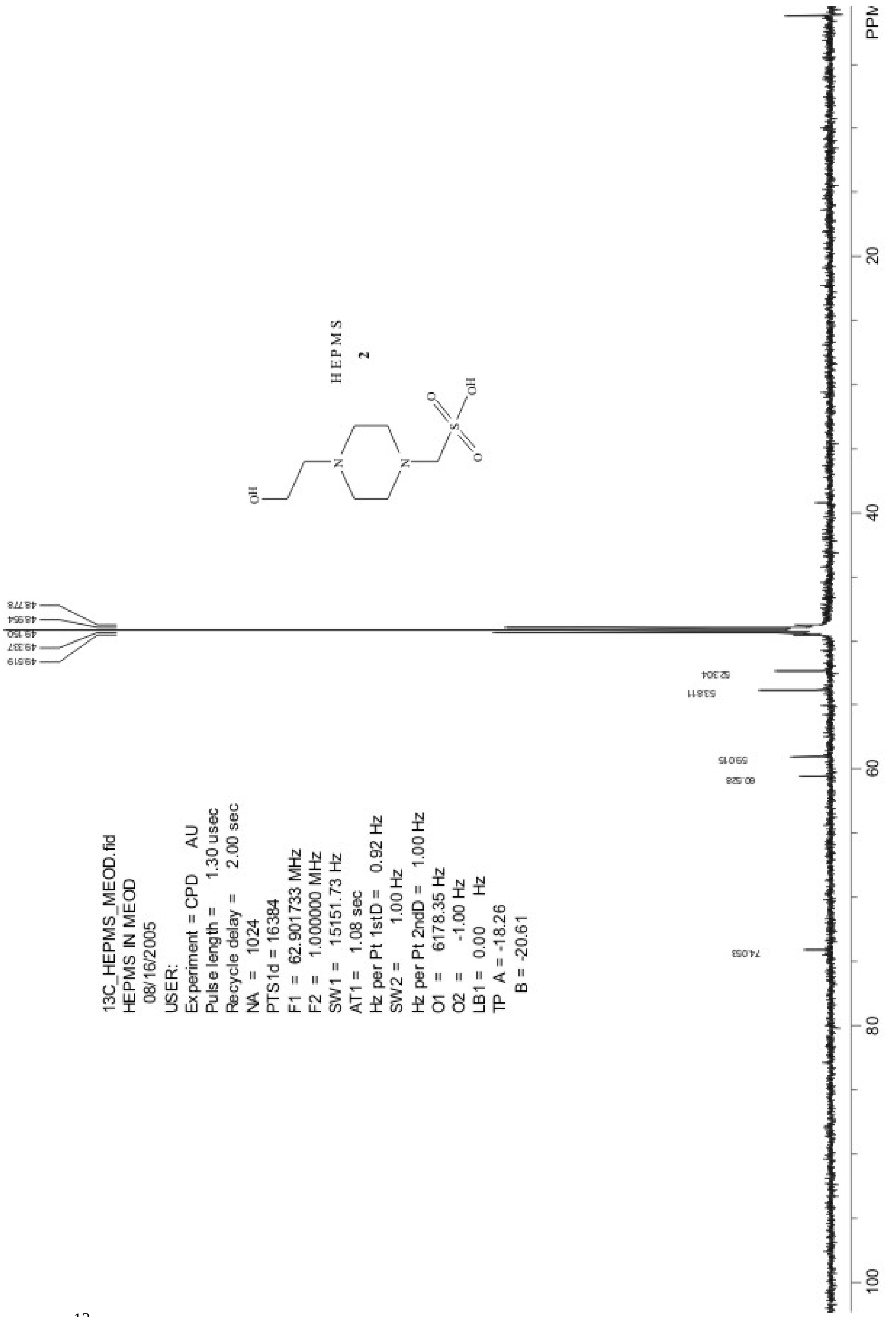

63 MHz ^{13}C NMR of HEPMS **2** in MeOD

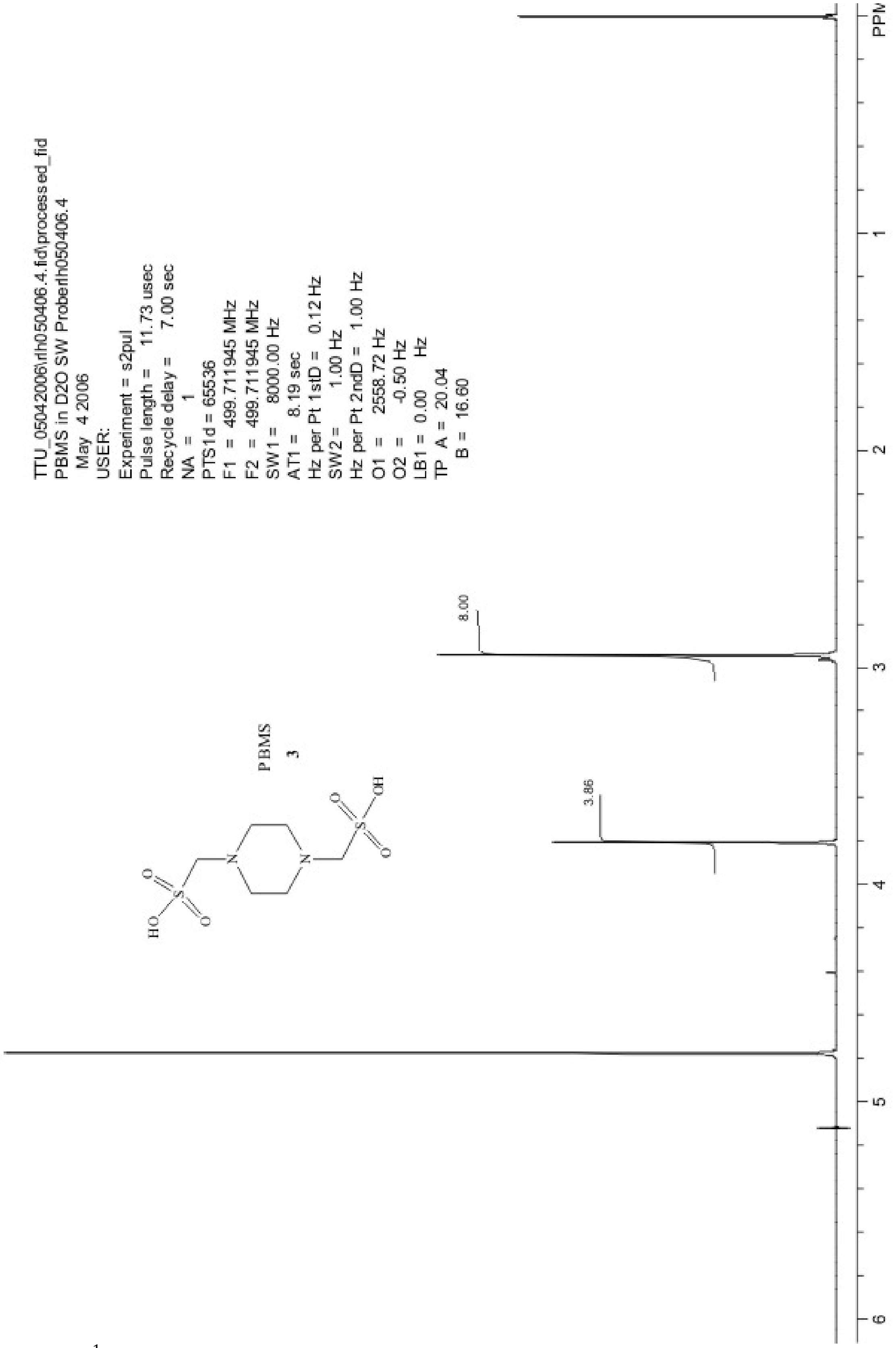

500 MHz ^{1}H NMR of PBMS **3** in D_2O with TSP reference

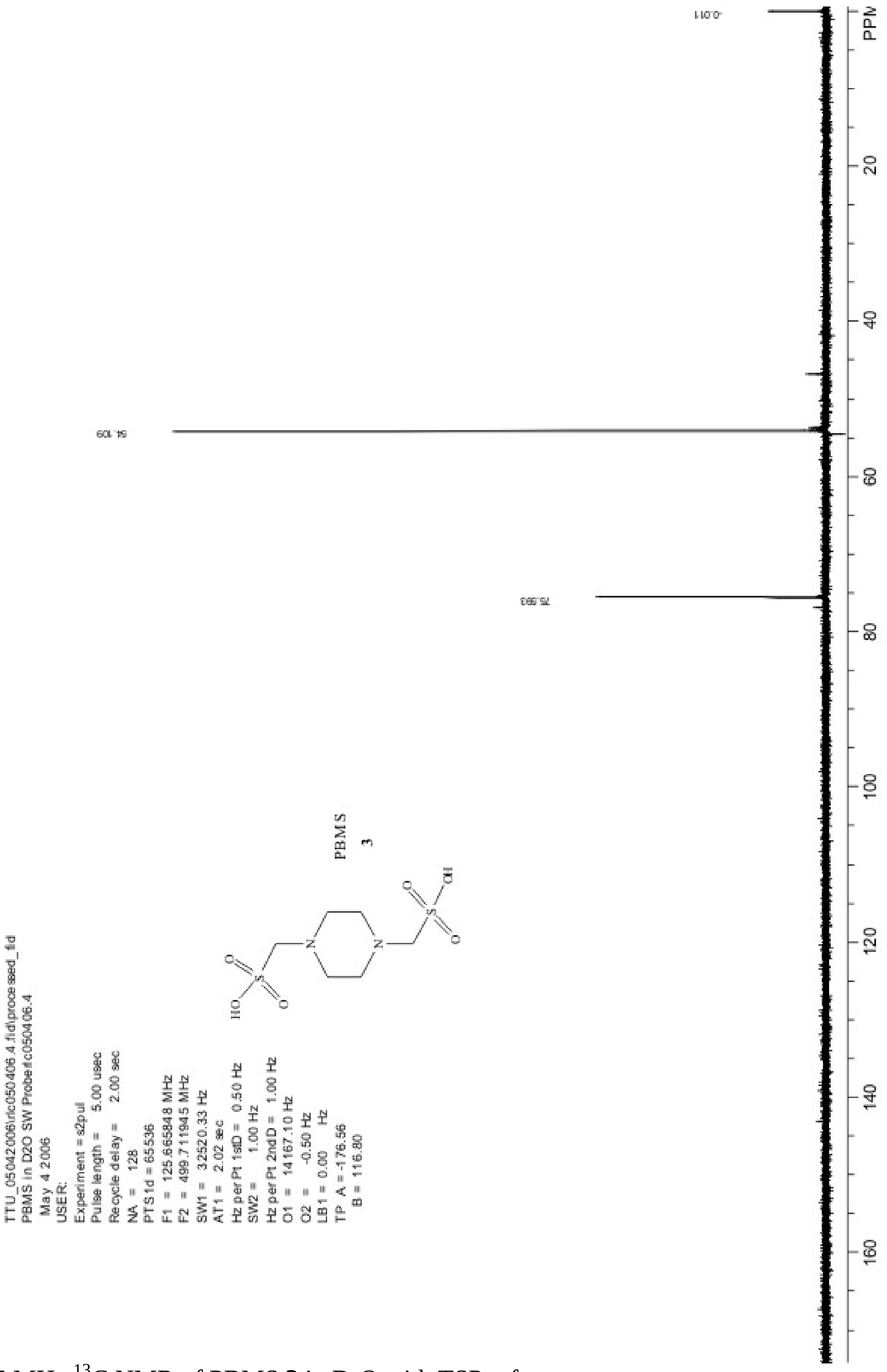

125 MHz ^{13}C NMR of PBMS **3** in D_2O with TSP reference

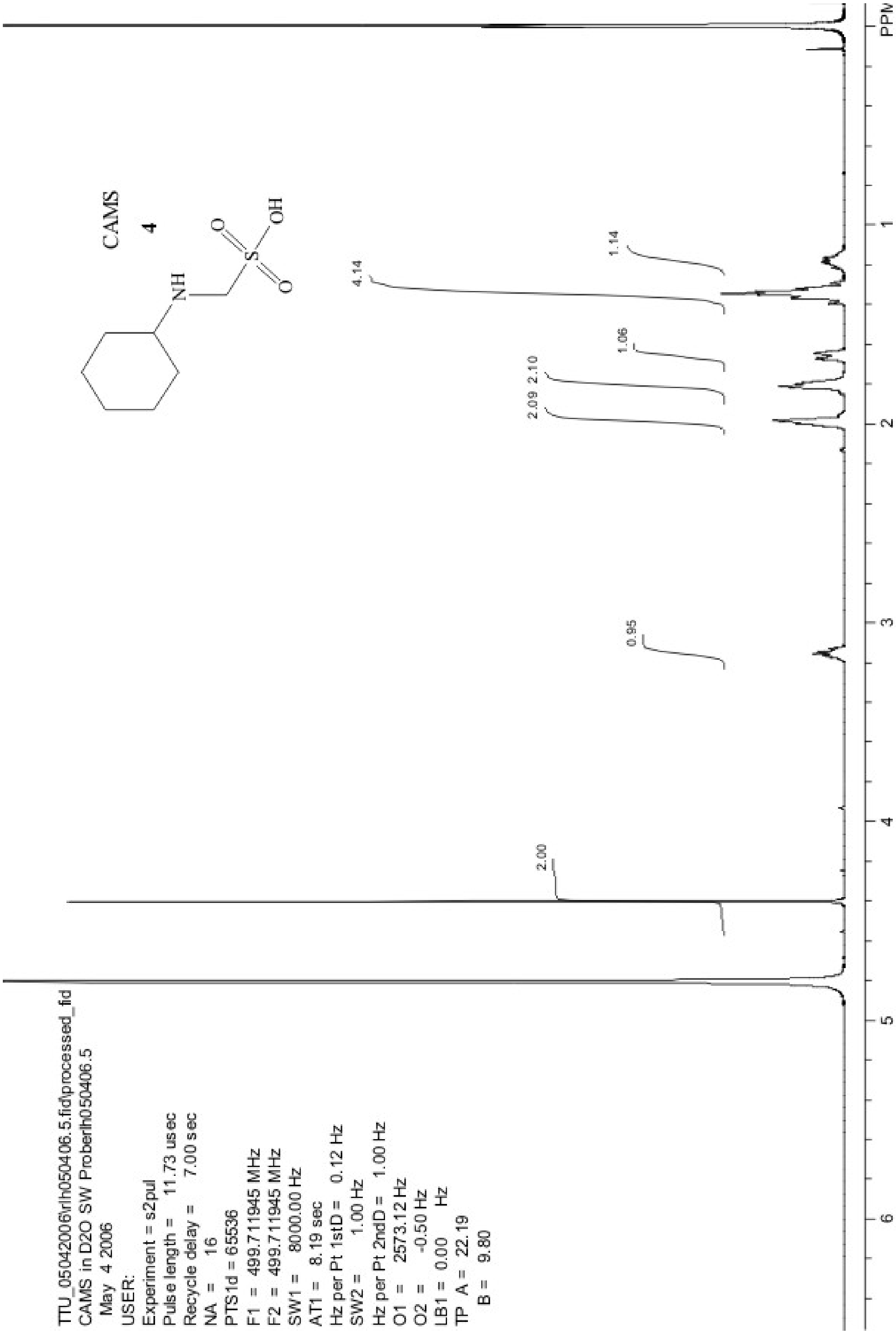

500 MHz ^{1}H NMR of CAMS **4** in D_2O with TSP reference

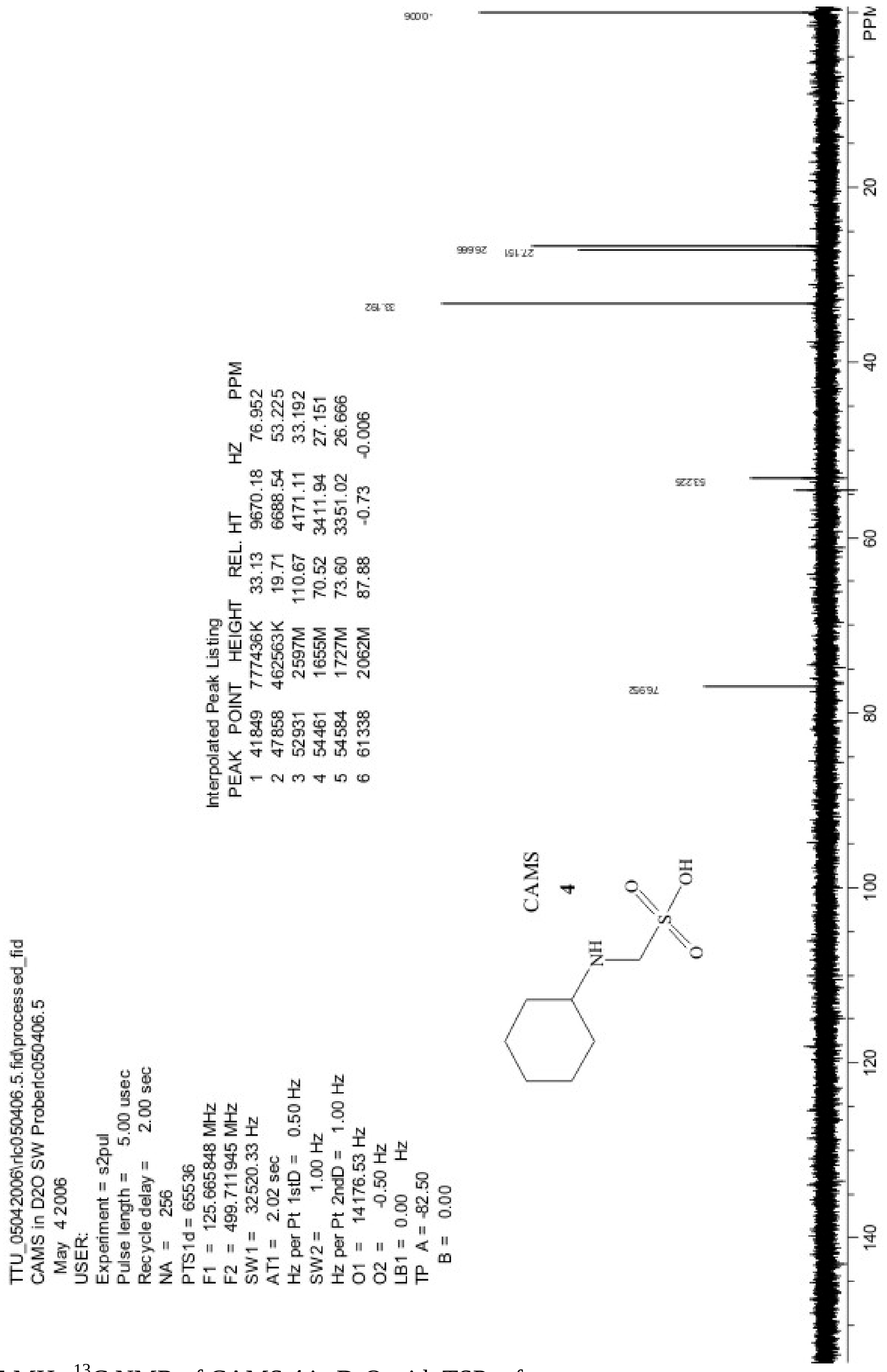

125 MHz ^{13}C NMR of CAMS **4** in D_2O with TSP reference

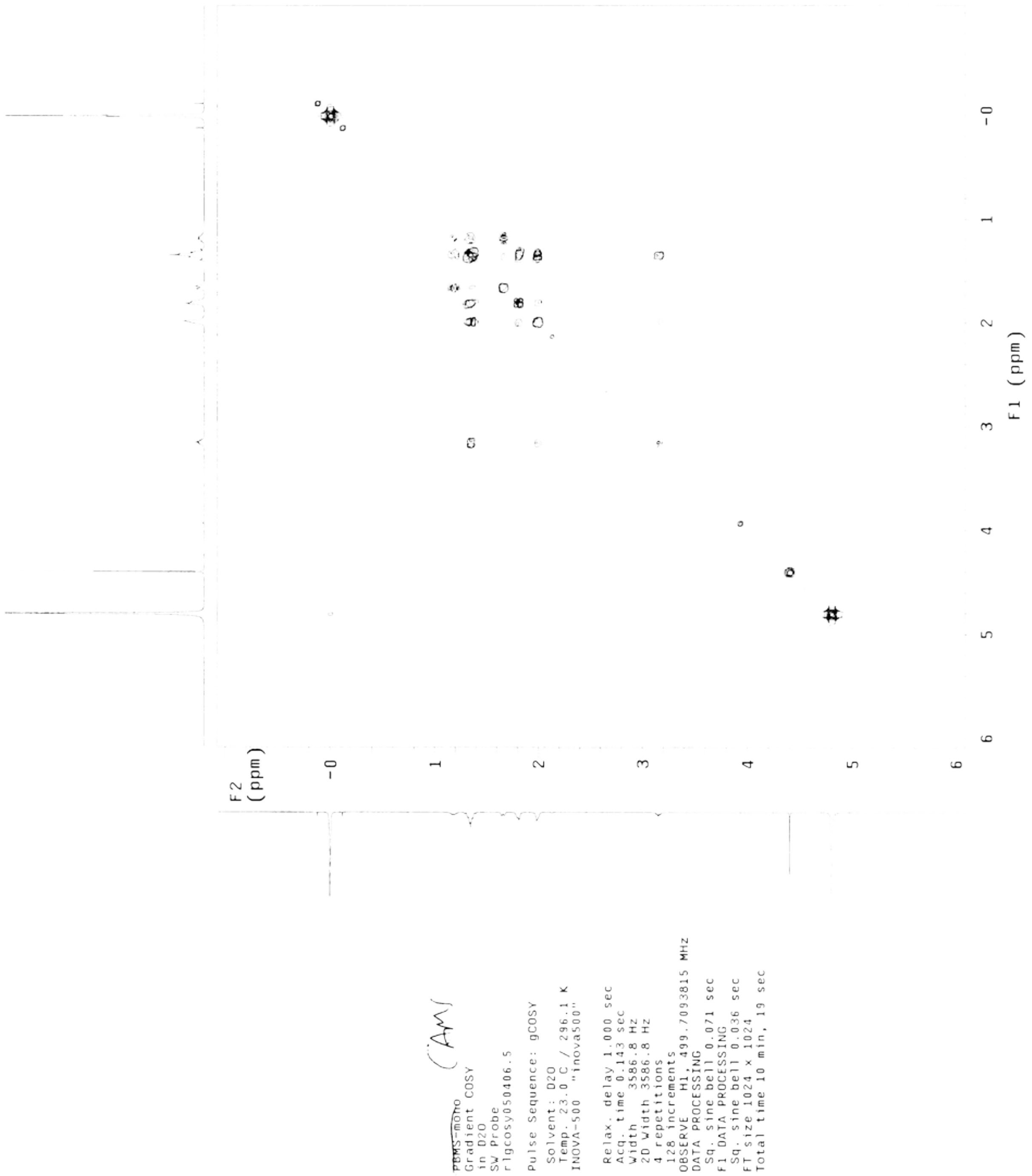

COSY of CAMS **4** in D_2O on 500 MHz NMR

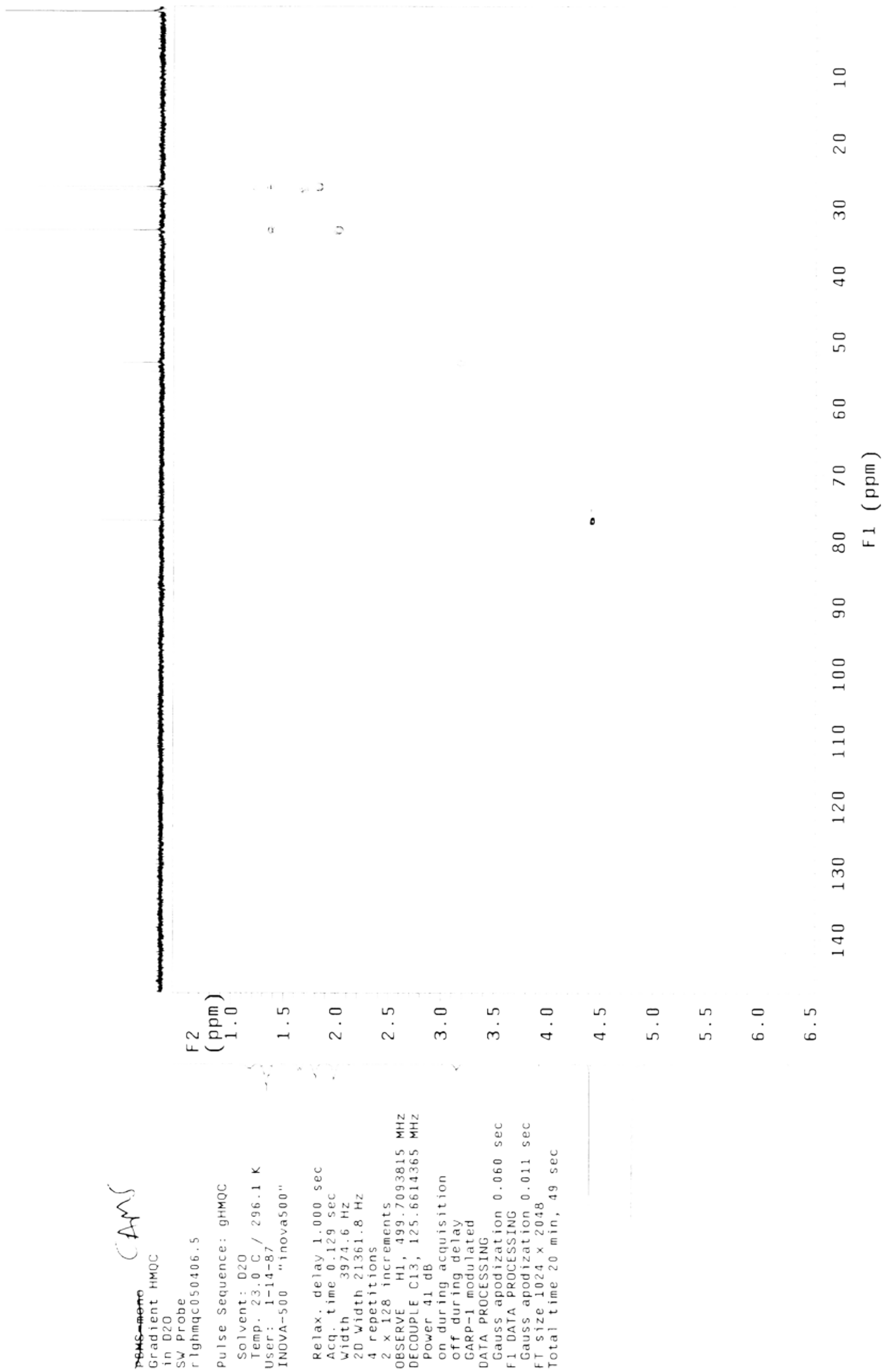

gHMQC of CAMS **4** in D_2O on 500 MHz NMR

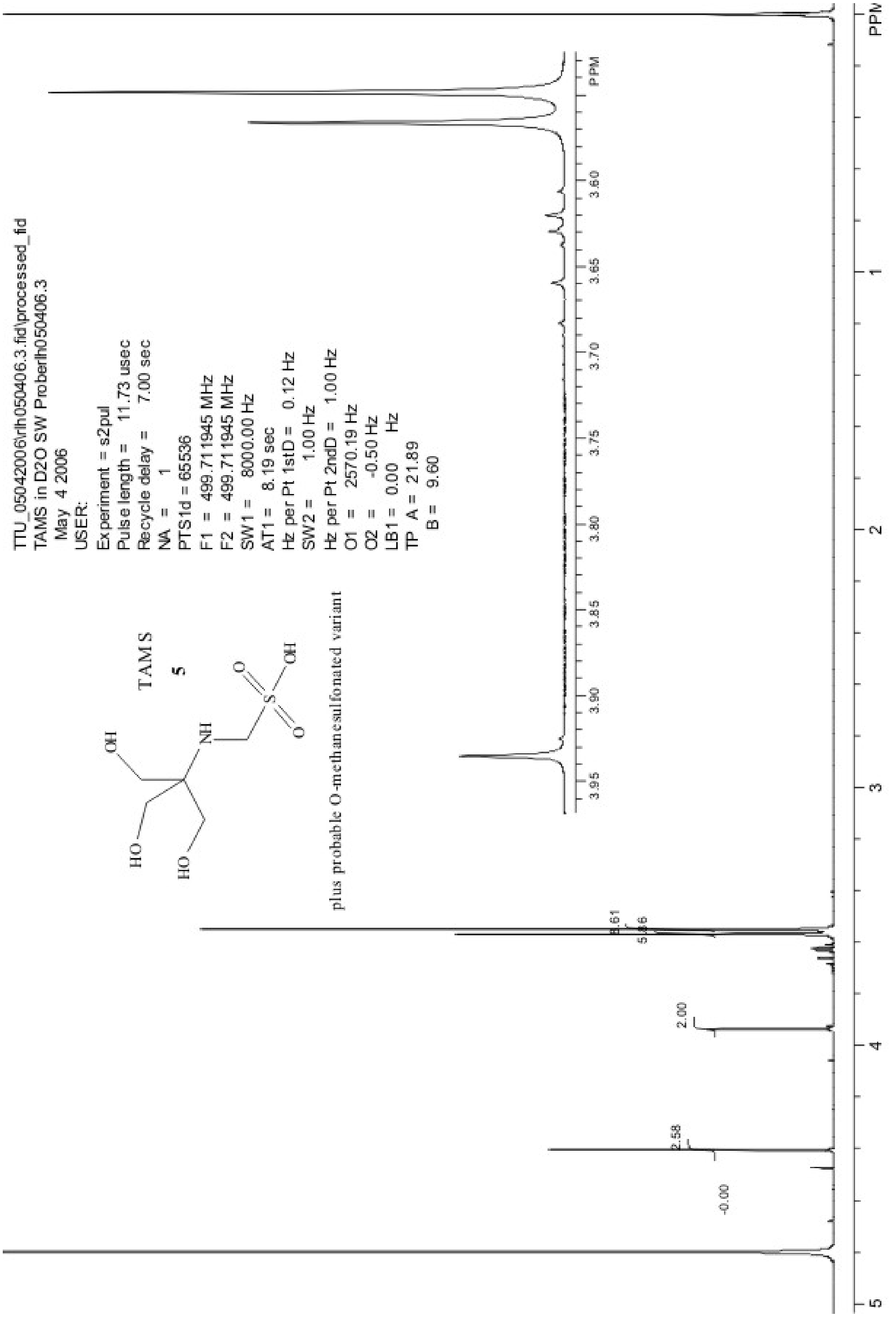

500 MHz ^{1}H NMR of TAMS **5** in D_2O with TSP reference (with byproduct)

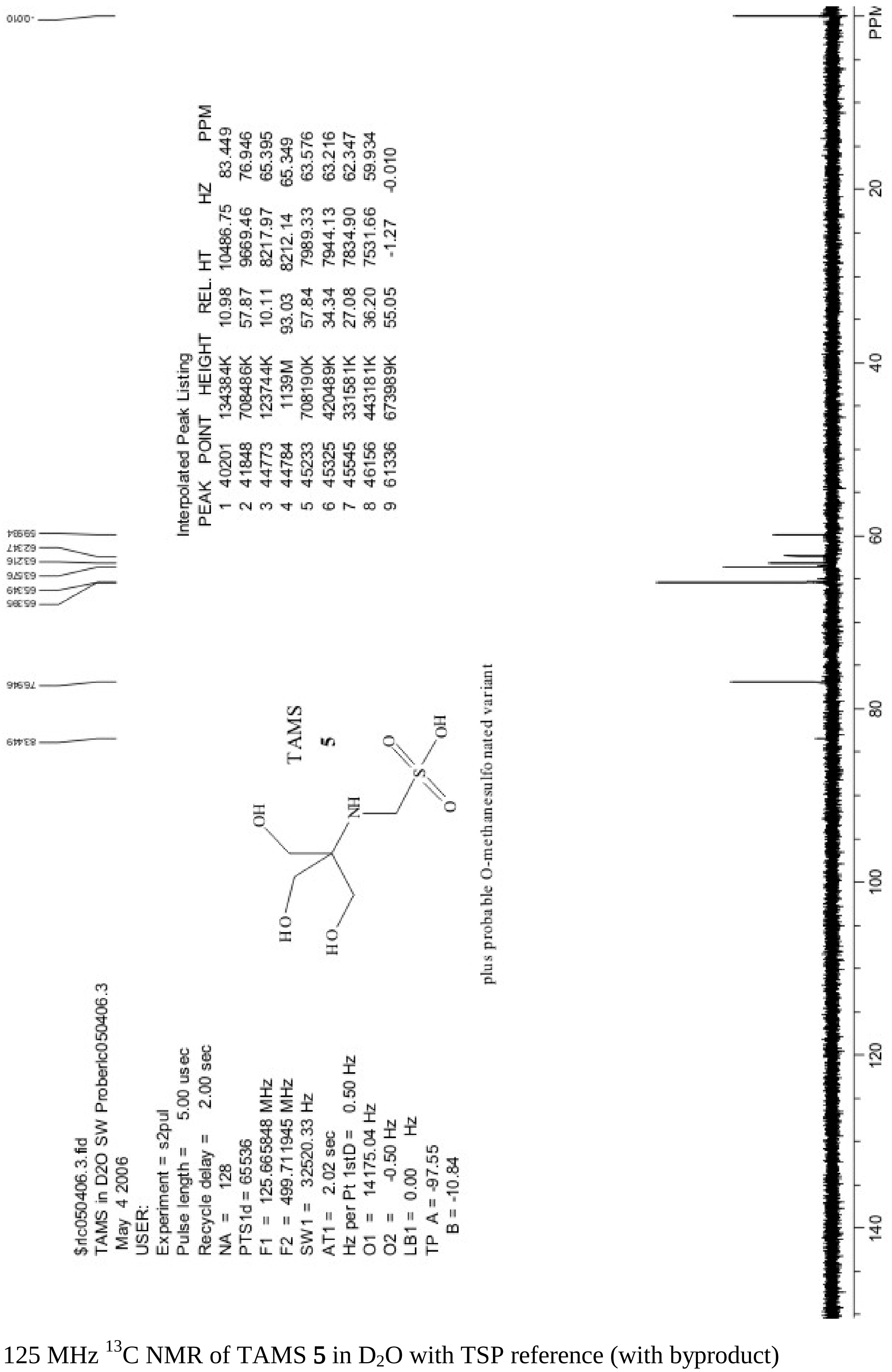

125 MHz ^{13}C NMR of TAMS **5** in D_2O with TSP reference (with byproduct)

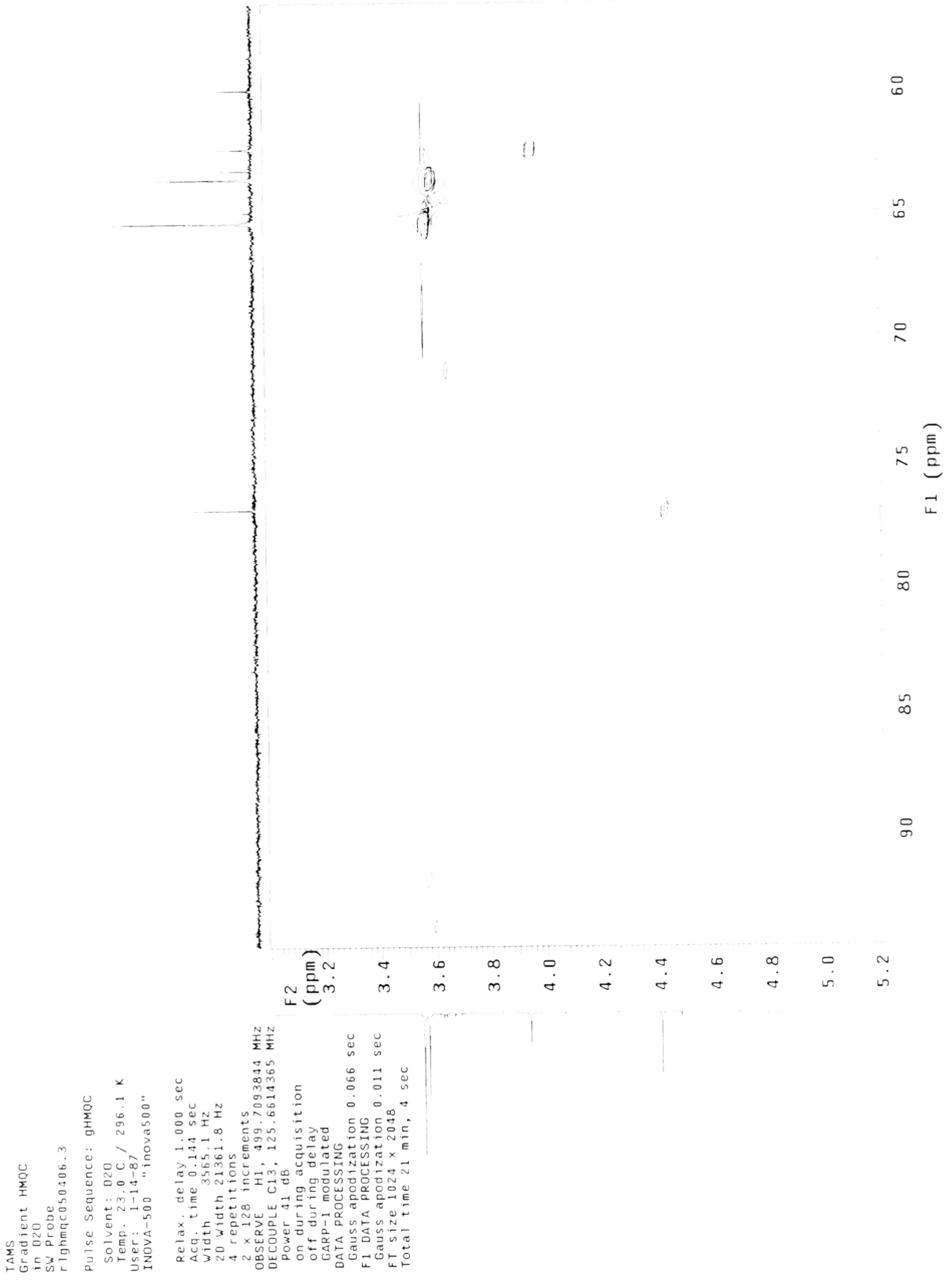

gHMQC of TAMS **5** sample in D_2O (with byproduct)

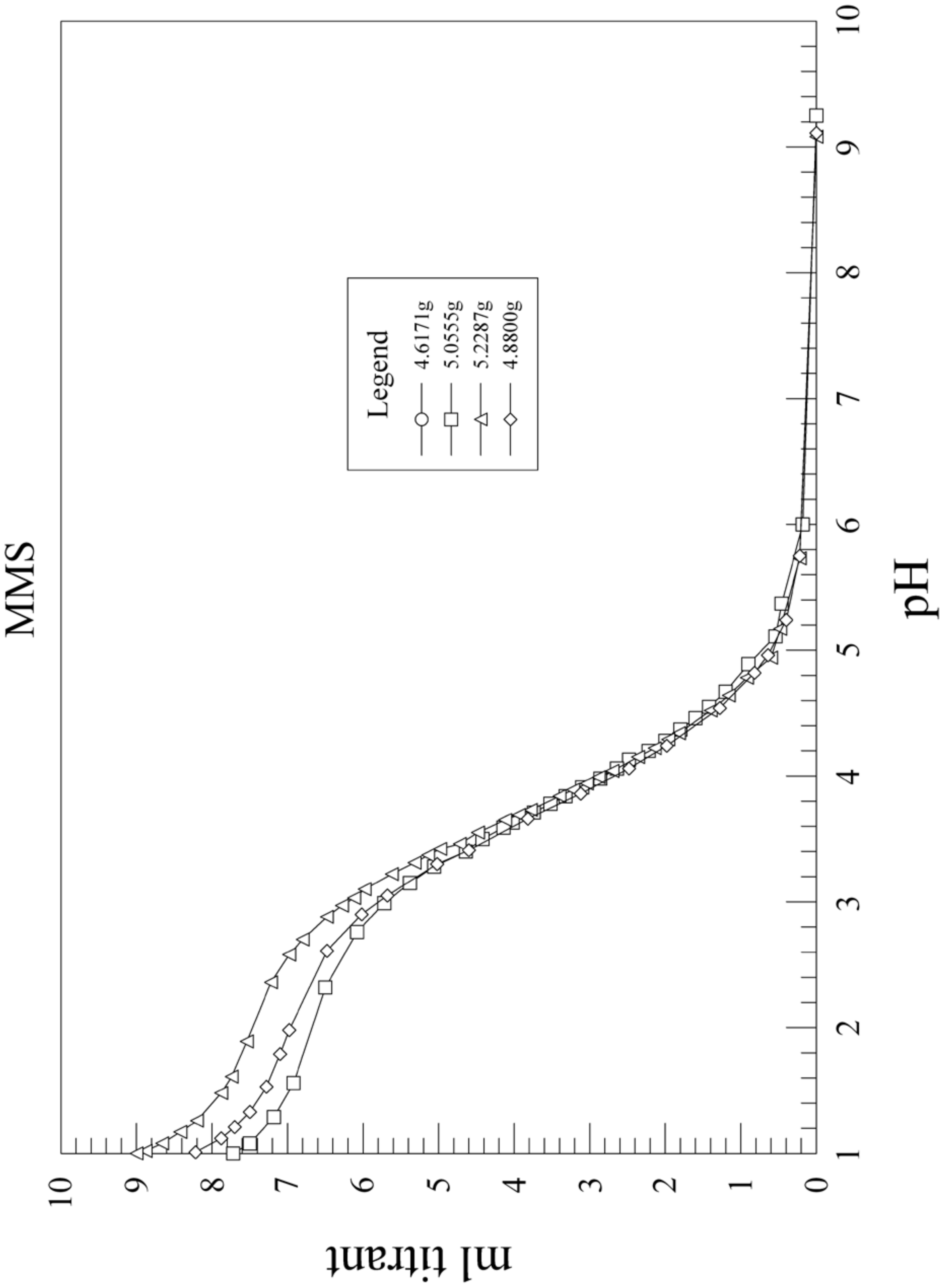

Titration curve of MMS **1** with 1M HCl

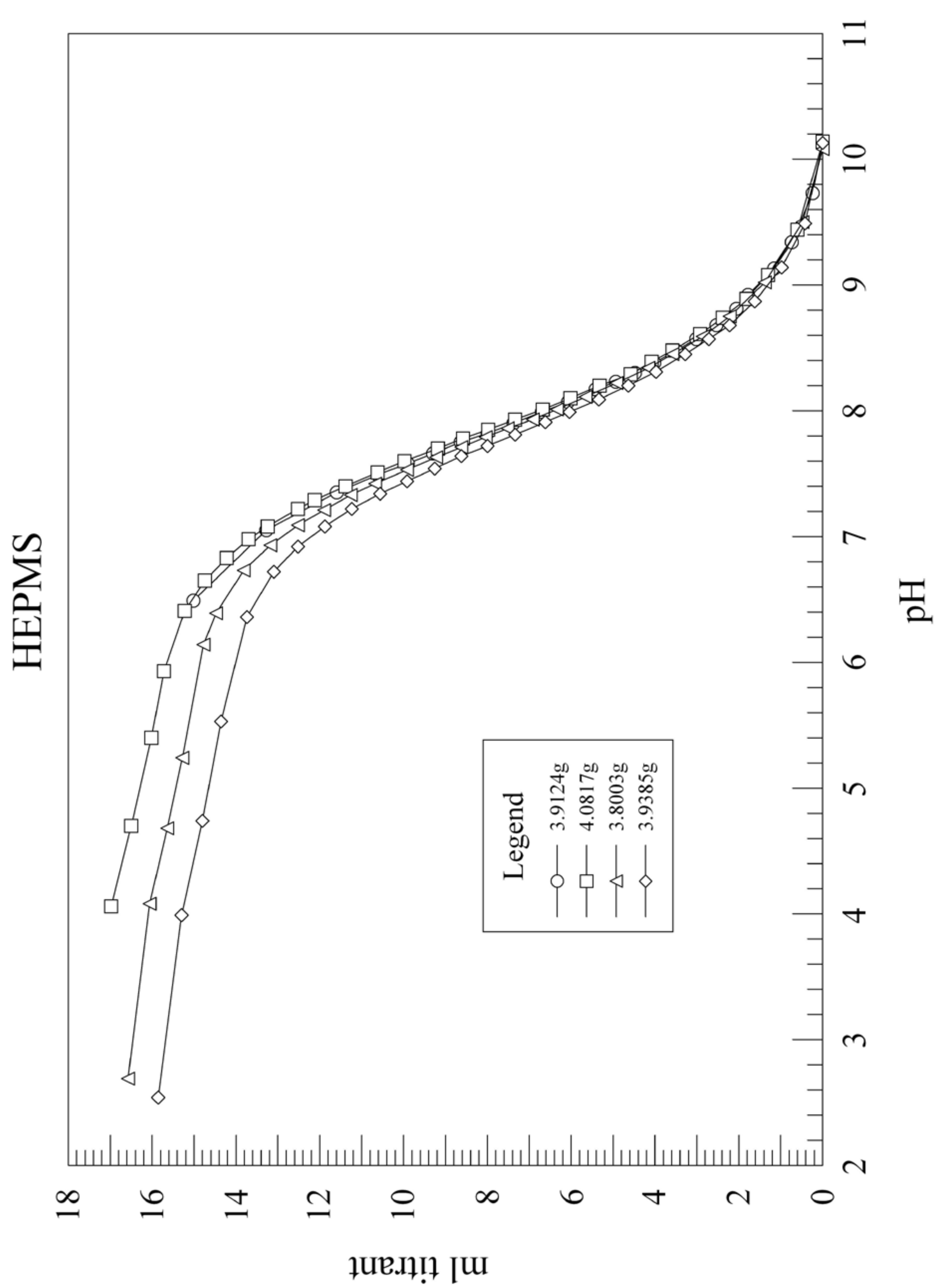

Titration curve of HEPMS **2** with 1M HCl

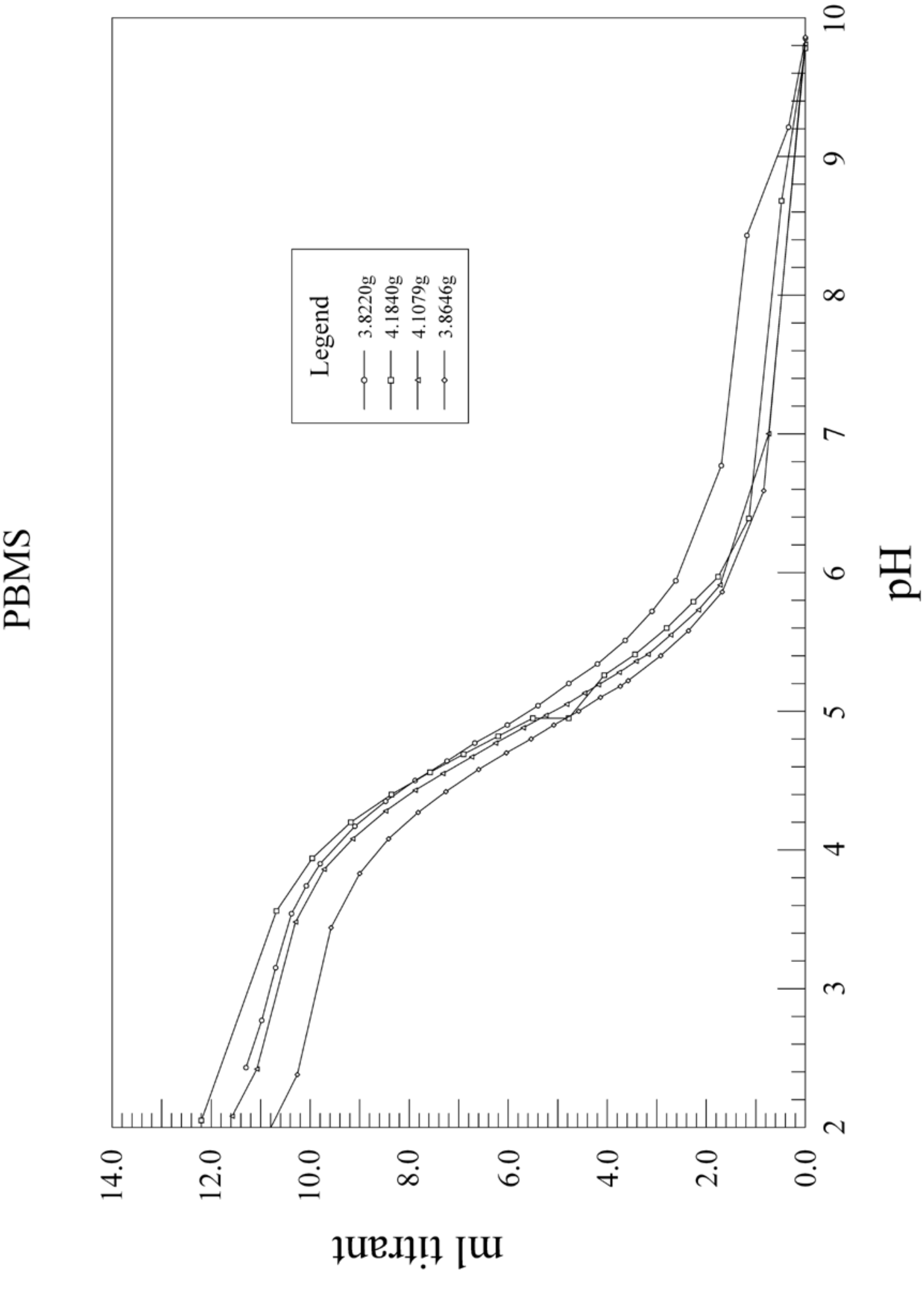

Titration curve of PBMS **3** with 1M HCl

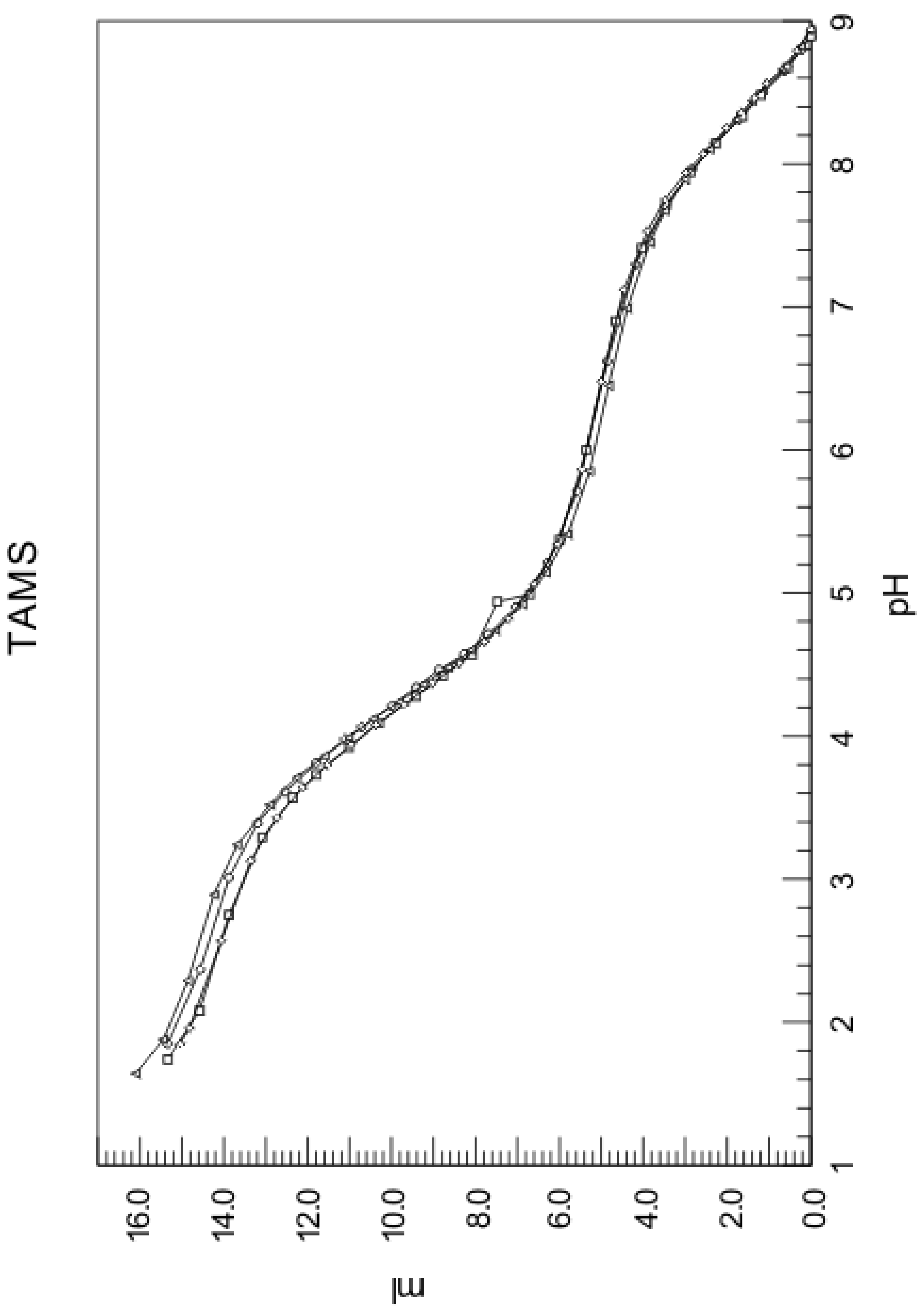

Titration curve of TAMS **5** (impure as synthesized) showing a probable pK_a of ≈ 4.1

Summary of Titration Data

	Number of samples	Avg pK_a	Std Error
MMS **1**	7	3.87	0.076
HEPMS **2**	4	7.88	0.069
PBMS **3**	4	4.90	0.049
CAMS **4**	3	6.92	0.56
*TAMS **5**	6	4.11	0.13

*TAMS is contaminated with unreacted Tris (observable in titration curves at pH 7–10)

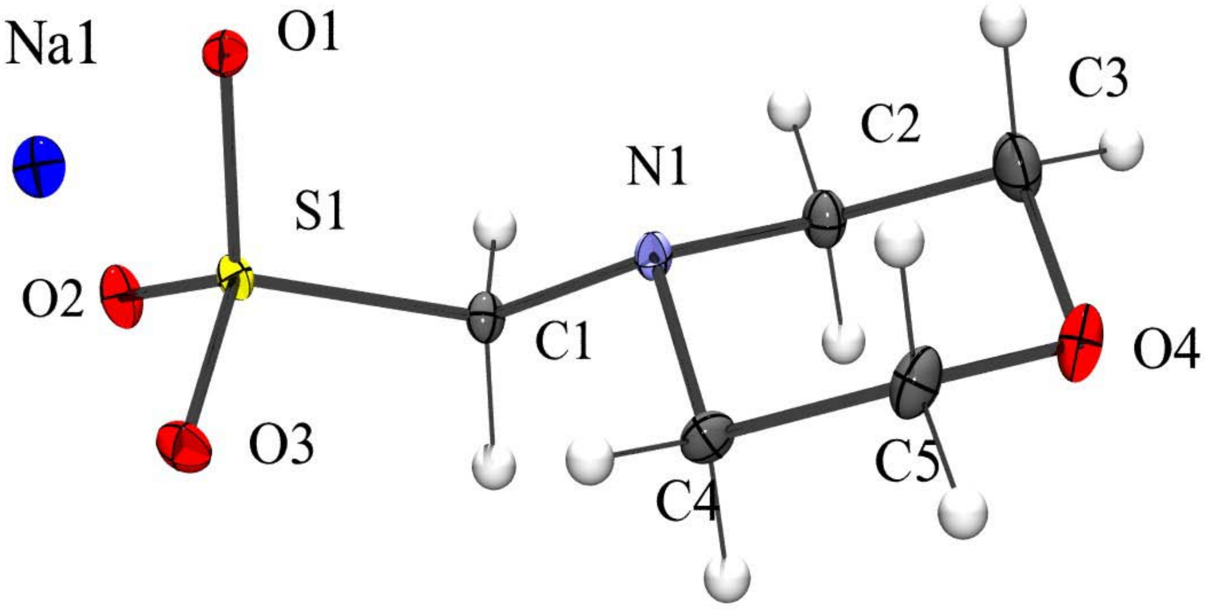

POV-Ray rendering of ORTEP3 figure for MMS **1** at 50% probability level

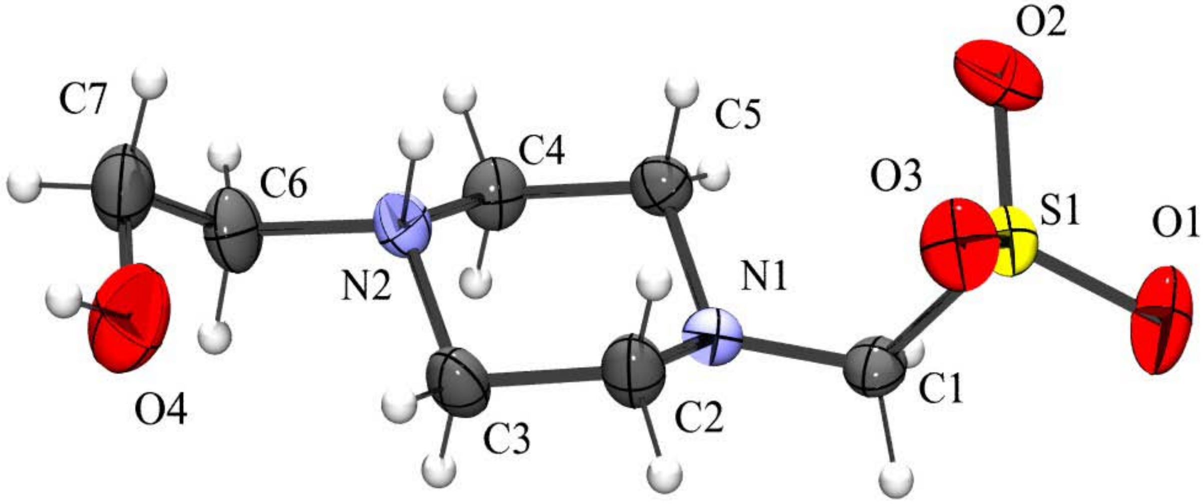

POV-Ray rendering of ORTEP3 figure for HEPMS **2** at 50% probability level
